# A targeted screening method for peripheral arterial disease: a pilot study

**DOI:** 10.1186/1757-1146-8-S2-O8

**Published:** 2015-09-22

**Authors:** Peta Craike, Vivienne Chuter

**Affiliations:** 1 Faculty of Health & Medicine, University of Newcastle, Ourimbah, NSW, 2258, Australia

**Keywords:** Vascular assessment, peripheral arterial disease, screening

## Background

Podiatrists play a central role in conducting non-invasive vascular foot assessment in the general population. Routine clinical assessment of lower limb arterial flow in at risk patients is essential in early detection and monitoring of the disease. There are a variety of clinical examinations available to clinicians in order to determine adequacy of blood supply to the lower extremity. However, recent research suggests that certain conditions, such as advanced age, diabetes, renal failure and medial arterial calcification can affect the accuracy of some of these examinations. Currently, there are no specific guidelines dictating the most appropriate methods of assessing lower limb vascular status for Podiatrists. The purpose of this study was to develop and validate a targeted vascular screening method for podiatrists.

## Methods

An extensive review of the literature was performed. Combined with recent research completed by the researchers, a targeted vascular screening method was developed. Two participant groups were recruited, a private practice group (N=31), and a public sector group (N=32). Two clinicians used the screening method to outline what assessment they would undertake. All non-invasive vascular testing along with colour duplex ultrasound was performed on all patients by a vascular ultrasonographer. Sensitivity and specificity of the methods chosen for detecting PAD were compared to the current American Heart Association (AHA) vascular screening guideline.

## Results

The targeted screening method demonstrated higher sensitivity (50%) than the AHA standard (33%) in the private participant group, and was equally as specific (88%). In participant group two, the targeted screening method was equally as sensitive to the AHA guideline (45.45%), but less specific (80.95% vs. 95.24%).

## Conclusions

The targeted screening method could be used as a guideline for podiatrists to make their vascular assessment more accurate, resulting in a decrease in the number of false negatives in patients with suspected PAD.

